# Amyand’s hernia with gangrenous perforated appendicitis and diffuse peritonitis—case report

**DOI:** 10.1093/jscr/rjaf913

**Published:** 2025-11-11

**Authors:** Radomir Gelevski, Gjorgji Jota, Blagica Krsteska, Vladimir Joksimovikj, Vesna Jovanovska Manevska, Bojan Trenchikj

**Affiliations:** Department of Surgery, General Hospital Kumanovo, 11 Noemvri bb, Kumanovo 1300, North Macedonia; University Clinic for Digestive Surgery, Ss Cyril and Methodius University, Vodnjanska bb, Skopje 1000, North Macedonia; Institute of Pathology, Medical Faculty, Ss Cyril and Methodius University, Vodnjanska bb, Skopje 1000, North Macedonia; University Clinic for Digestive Surgery, Ss Cyril and Methodius University, Vodnjanska bb, Skopje 1000, North Macedonia; Department of Surgery, General Hospital Kumanovo, 11 Noemvri bb, Kumanovo 1300, North Macedonia; Department of Surgery, General Hospital Kumanovo, 11 Noemvri bb, Kumanovo 1300, North Macedonia

**Keywords:** Amyand’s hernia, acute appendicitis, perforated appendix, inguinal hernia, peritonitis

## Abstract

Amyand’s hernia, the presence of the vermiform appendix within an inguinal hernia sac, is a rare clinical entity (0.2%–1.7% of inguinal hernias). Acute appendicitis within the sac is rarer still, occurring in only 0.1% of cases. We describe a male patient presenting with an incarcerated right inguinal hernia, who developed diffuse peritonitis after manual reduction. Laparotomy revealed gangrenous perforated appendicitis with purulent peritonitis. Appendectomy, partial omentectomy, and peritoneal lavage were performed. The patient recovered uneventfully. Amyand’s hernia complicated by perforated appendicitis is an exceptional surgical emergency. Early recognition and prompt surgical management are crucial for favorable outcomes.

## Introduction

Amyand’s hernia, first described in 1735 by Claudius Amyand, refers to the presence of the appendix within an inguinal hernia sac [1]. It is a rare condition, found in ~0.2%–1.7% of inguinal hernias [1, 2]. Acute appendicitis occurring inside the hernia sac is even rarer, with an incidence of 0.07%–0.13% of all appendicitis cases [2, 3]. The condition may mimic an incarcerated or strangulated hernia and is often only diagnosed intraoperatively due to its nonspecific presentation. When perforation occurs within the hernia sac, the risk of peritonitis increases substantially, representing a rare but serious surgical emergency [3, 4].

Acute appendicitis in a hernial sac can occur due to extraluminal compression of the appendix at the narrow hernia neck, leading to compromised blood flow, ischemia, and subsequent bacterial invasion [3, 5]. Hernial sac appendicitis is most commonly described in inguinal hernias (Amyand’s hernia), but has also been reported in femoral (De Garengeot’s hernia) and umbilical hernias [4]. Because the appendix is trapped outside its usual anatomical location, inflammatory progression may be more rapid, increasing the risk of gangrene and perforation.

Routine laboratory markers such as leukocytosis and C-reactive protein (CRP) are widely used to support diagnosis of appendicitis, but they lack specificity in differentiating uncomplicated from complicated disease. Recently, the neutrophil-to-lymphocyte ratio (NLR) has gained attention as a marker of systemic inflammation and predictor of complicated appendicitis [5].

Hyperbilirubinemia has also emerged as a clinically useful adjunct in risk stratification. Several studies demonstrate that elevated serum bilirubin correlates with gangrenous or perforated appendicitis, with a specificity higher than that of white blood cell count (WBC) and CRP. The pathophysiology is attributed to endotoxin-induced hemolysis and cytokine-mediated impairment of hepatocellular excretion, leading to cholestasis [5].

Management of Amyand’s hernia is guided by the Losanoff–Basson classification, which remains the most widely accepted system [6]. It categorizes Amyand’s hernia into four subtypes:

- Type 1: normal appendix within hernia sac → reduction and mesh hernia repair; appendectomy only in young patients.
- Type 2: acute appendicitis within hernia sac, no sepsis → appendectomy through hernia sac, primary repair without mesh.
- Type 3: acute appendicitis with peritonitis or abdominal wall sepsis → laparotomy, appendectomy, peritoneal lavage, hernia repair without mesh or delayed repair.
- Type 4: Amyand’s hernia with concurrent unrelated intra-abdominal pathology → individualized management according to findings.

## Case presentation

A male patient presented to the emergency department with 12 hours of abdominal pain, initially epigastric, later localizing to the right inguinal region, accompanied by fever, anorexia, and nausea.

Examination revealed an incarcerated right inguinal hernia, firm and tender with normal skin coloration. Manual reduction was achieved, but the patient subsequently developed signs of diffuse peritonitis. At the moment of establishing indication for laparotomy, acute perforated appendicitis was not considered among the differential diagnoses, as the clinical impression was dominated by the acute abdomen that developed immediately after manual reduction. A contrast-enhanced abdominal CT scan was not obtained because the acute abdomen was identified after the manual reduction of the incarcerated hernia, and the leading suspicion was on bowel perforation rather than appendicitis.

The laboratory findings were as follows: WBC 18 × 10^9^/l, neutrophils 15.5 × 10^9^/l, NLR 9.06, CRP 26.94 mg/l, sodium 136 mmol/l, total bilirubin 37.4 μmol/l, and procalcitonin 0.1 ng/ml. Elevated bilirubin and NLR were consistent with complicated appendicitis [5].

Surgical exploration with an emergency laparotomy revealed a gangrenous appendix with perforation at the base inside the hernia sac, with diffuse purulent peritonitis. Appendectomy with secure stump closure, partial omentectomy, and extensive peritoneal lavage was performed.

**Figure 1:**
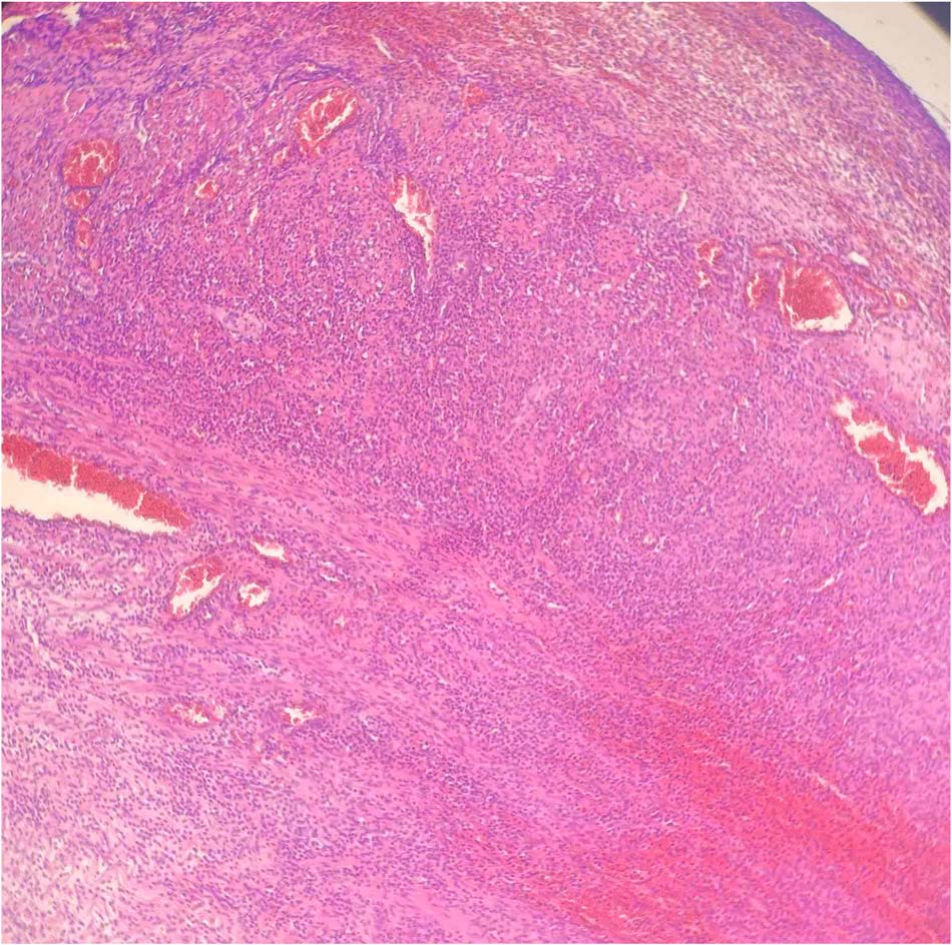
Hematoxylin Eosin (HE) stain 100× transmural acute inflammation with peritonitis.

**Figure 2:**
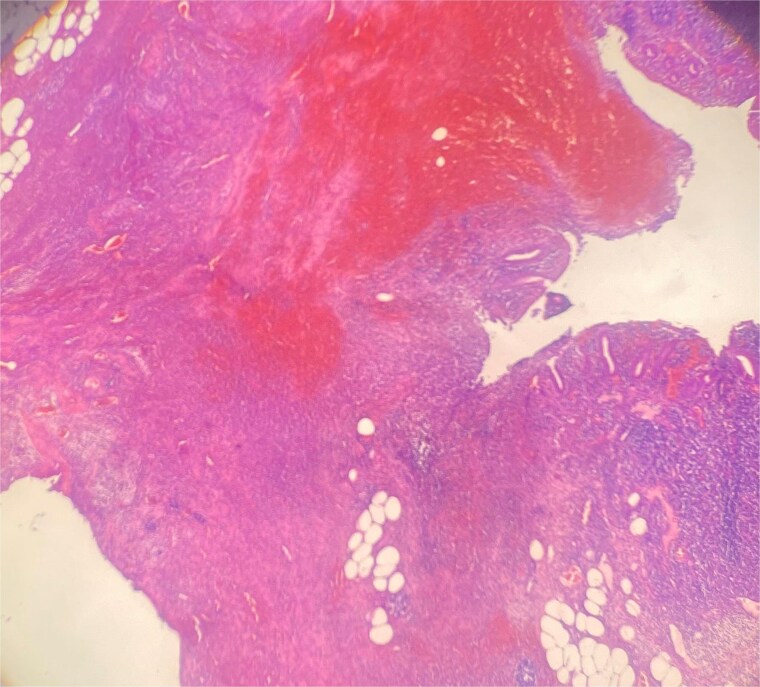
HE 40× wall perforation with hemorrhage and inflammation.

Histopathology confirmed gangrenous appendicitis with perforation (Figs 1 and 2). The postoperative course was uneventful; peristalsis returned on Day 2, with meticulous follow-up of laboratory parameters, gradual mobilization and restauration of bowel function from Days 3–7, and the patient was discharged on Day 7 in good condition.

## Discussion

Amyand’s hernia is rare, accounting for <1% of inguinal hernias, with acute appendicitis inside the hernia sac occurring in only 0.1% of cases [1–3]. The condition may remain clinically silent or mimic an incarcerated hernia, which often leads to delayed or intraoperative diagnosis. When perforation and peritonitis occur, the condition represents a surgical emergency with historically high morbidity and mortality [4, 7].

Manual reduction (“taxis”) is sometimes attempted in emergency settings to relieve incarceration, but it carries significant risks. Reduction en masse occurs when diseased viscera are pushed into the abdominal cavity without true relief of obstruction, masking the underlying pathology [4]. In cases of Amyand’s hernia, reduction of an inflamed or perforated appendix into the peritoneal cavity can lead to delayed recognition of peritonitis and worsening sepsis [5]. Our case similarly illustrates this risk: after successful disincarceration, the patient rapidly developed diffuse peritonitis due to previously unrecognized perforated appendicitis. This underscores the need for vigilant postreduction monitoring and a low threshold for laparotomy if signs of systemic deterioration appear.

Traditional markers such as leukocytosis and CRP are helpful but nonspecific for complicated appendicitis. Recent evidence suggests that the NLR and CRP-to-albumin ratio (CAR) may better predict disease severity [6]. In our patient, both the NLR (9.06) and CAR (0.68) were elevated, consistent with systemic inflammation and complicated disease.

Hyperbilirubinemia has gained recognition as a prognostic marker for complicated and perforated appendicitis. Multiple studies and meta-analyses confirm that elevated total bilirubin (>20–25 μmol/l) correlates strongly with gangrenous or perforated appendicitis. The pathophysiology is thought to involve endotoxemia-induced hemolysis, impaired hepatic uptake of bilirubin, and cytokine-mediated cholestasis [5].

In our case, total bilirubin was markedly elevated at 37.4 μmol/l, which correlated with the intraoperative finding of gangrenous perforated appendicitis and diffuse peritonitis. This supports growing evidence that bilirubin may serve as an adjunct marker to risk-stratify patients preoperatively and anticipate complicated intra-abdominal pathology [5].

The Losanoff–Basson classification provides a practical framework for management [6]. Our case corresponded to Type 3, requiring laparotomy, appendectomy, and lavage without mesh hernia repair in the contaminated setting. The literature consistently advises against mesh placement in the presence of sepsis to avoid prosthetic infection.

Recent reports of Amyand’s hernia with perforated appendicitis describe similar approaches, with appendectomy, lavage, and staged hernia repair yielding good outcomes [6, 7]. Despite historically reported mortality rates of up to 30% in complicated cases, early recognition and prompt surgical intervention significantly improve prognosis.

## Conclusion

Amyand’s hernia with perforated appendicitis and diffuse peritonitis is a rare but life-threatening condition. Manual reduction may obscure pathology, delaying diagnosis. Surgeons should remain alert to postreduction deterioration. Early laparotomy, appendectomy, and lavage, with delayed hernia repair, remain the gold standard for management.

## References

[1] Sharma H, Gupta A, Shekhawat NS, et al. Amyand’s hernia: a report of 18 consecutive patients over a 15-year period. Hernia 2007;11:31–5. 17001453 10.1007/s10029-006-0153-8

[2] Michalinos A, Moris D, Vernadakis S. Amyand’s hernia: a review. Am J Surg 2014;207:989–95. 10.1016/j.amjsurg.2013.07.043 24280148

[3] D’Alia C, Lo Schiavo MG, Tonante A, et al. Amyand’s hernia: case report and review of the literature. Hernia 2003;7:89–91. 12820031 10.1007/s10029-002-0098-5

[4] Cross A, Yonkus J, Turay D, et al. Reduction en masse of incarcerated inguinal hernia: a case report. Int J Surg Case Reports 2024;123:110222. 10.1016/j.ijscr.2024.110222 PMC11409008 39245012

[5] Giordano S, Pääkkönen M, Salminen P, et al. Elevated serum bilirubin in assessing the likelihood of perforation in acute appendicitis: a diagnostic meta-analysis. Int J Surg 2013;11:795–800. 10.1016/j.ijsu.2013.05.029 23732757

[6] Losanoff JE, Basson MD. Amyand hernia: what lies beneath—a proposed classification scheme to determine management. Am Surg 2007;73:1288–90. 10.1177/000313480707301221 18186392

[7] Ali SM, Malik KA, Al-Qadhi H. Amyand’s hernia: study of four cases and literature review. Sultan Qaboos Univ Med Jl 2012;12:232–6. 10.12816/0003119 PMC3327573 22548145

